# Memoir on the Loss of the Teeth

**Published:** 1856-07

**Authors:** 

**Affiliations:** Paris


### ARTICLE X.

*Memoir on the Loss of the Teeth.* By Rossi, Paris, 1852.

Continued from page 301.

### 3.—Arrangement of the Teeth.

When the second dentition has not been regular; when a skillful hand has not been called to remedy errors which nature often commits, and which it is always possible to prevent or correct, this irregularity in the arrangement of the teeth may be the origin of their decomposition.

The teeth are often within or without the usual circle, instead of being regularly placed at the side of each other. These haunts for food, resulting from this vicious arrangement, become laboratories in which destructive acids are formed. We will state here briefly that teeth having cavities hollowed out on their approximal surfaces become dangerous neighbors, because they hold destructive agents in contact with the teeth which they touch.

We have nothing further to say about the arrangement of the teeth and of the importance of this predisposing cause; when it favors the sojourn of aliments, it thus becomes a means of destruction. The natural separation of the teeth is a fortunate distribution, because it does not permit the accumulation of alimentary detritus.

### 4.—Traumatic Lesions.

They may render the destruction of the teeth easier by depriving them of their covering of enamel, and above all by giving them a form which retains aliments. But as this cause is rare, and as moreover it seldom occurs except upon the molars already attacked, although it may not be apparent, we only mention it to render our list complete.

### SUMMARY.

We have completed the study of the causes which destroy the teeth. We have made every effort to avoid useless prolixity in the discussion, or in the examination of facts, in order that the mind might embrace, without being distracted, and at a single glance, so to speak, the whole of the theory. We desired above all to be clear and precise. The reader only can decide whether we have succeeded.

We will sum up what precedes, by a few general considerations.

We have just seen that the destruction of the teeth attributed to a pathological cause termed *caries*, is due only, in reality, to a chemical action of the acids which exist in the humors of the subject, which arise from the putrid or acetic fermentation of aliments, or introduced into the mouth already formed.

That the teeth, an epidermic product, are not penetrated by a single vessel, by a single nerve, and cannot modify themselves physiologically in such a manner as to be exposed to a morbid condition.

That persons from a superficial observation have readily been caused to think for a time that the molars, in particular, sometimes decayed from within outward, for nothing exteriorly indicated the presence of even a considerable cavity already existing in the interior.

That, however, a more minute, attentive examination will always discover an opening, a funnel filling the alimentary detritus upon the ivory through the layer of enamel.

That the interdentiary spaces favored the accumulation and sojourn of this detritus.

That it was there in fact that the teeth were usually attacked.

That the form of the teeth, as well as their arrangement, was sometimes a condition of great importance.

That the action of predisposing causes should be regarded as very important in this process of destruction.

That the lymphatic temperament predisposes to their loss, as the ossification of the teeth is less compact and resisting.

That the union of all these causes or the action of a few only, concurred to their disorganization.

There still remains for us to give a reason for certain facts which the pathological theory cannot explain. The study which we have just completed furnishes us the means, and the influence of the *unique cause*, the only one which destroys the teeth, will be more clearly demonstrated from it. We will set forth these facts under the form of propositions which we will discuss one by one.

CONSEQUENT PROPOSITIONS.

I.—The superior incisors decay; the same teeth of the lower jaw remain sound.

II.—The superior central incisors of an arched form often decompose on their external surface.

III.—The superior lateral incisors are sometimes attacked on their internal surface.

IV.—The superior canines resist oftener than the incisors and superior bicuspids.

V.—The superior bicuspids are often destroyed when all the other teeth remain perfectly sound.

VI.—The first molars are oftener destroyed than the other molars, the wisdom tooth excepted.*

* The bicuspids are classed among the molars by our author.—TRANS.

VII.—The molars decompose by their approximal surface, the superiors generally in mature age having remained sound until then.

VIII.—The inferior molars are sometimes destroyed in aged persons by their external surfaces and at the neck.

IX.—The superior wisdom teeth are often decomposed by their external surfaces, the inferiors by their grinding surfaces.

X.—The inferior wisdom tooth is oftener destroyed than the superior.

WE WILL DISCUSS THESE PROPOSITIONS.

I.—The superior incisors decay, the same teeth on the lower jaw remain sound.

The buccal mucus is in constant contact with the superior incisors; the saliva with the inferior incisors. The buccal mucus is acid, very acid under the influence of certain temperaments, of some morbid conditions. The saliva on the contrary is alkaline. Alimentary detritus readily remains in the interstitial spaces of the superior teeth while it is constantly diluted and carried away below and anteriorly, for this is the most dependent part.

The mucus then acts upon the superior, the saliva upon the inferior incisors, the former to destroy, the latter to preserve. Alimentary detritus may exhaust its action on the upper teeth, it has none on the lower, for it cannot sojourn there but a short time, and not long enough to ferment. This is the cause of this exception, apparently so strange.

II.—The central incisors of an arched form often decompose on their labial surfaces near the neck. We have exposed in detail the causes of this decomposition, in treating of the form of the teeth; we refer the reader to it. (See page 79.)

III.—The superior lateral incisors are sometimes attacked on their posterior surfaces. There is often upon this surface, a conical depression or deep crevice in the enamel. It is an anomalous form frequently met with. As there is in this case no contiguity but only an imperfect contiguity of the enamel, the imbibition of fluids to the dentine is favored by the phenomenon of capillary attraction.

IV.—The superior canines resist better than the incisors or the superior bicuspids.

This is a problem of form. Their approximal surfaces are rounded, in place of being flat, like the incisors and bicuspids, they touch their neighbors only at one point, the sojourn of alimentary detritus in the spaces which separate them is thus rendered more difficult.

V.—The superior bicuspids are often attacked or destroyed when all the other teeth are pefectly intact.

This proposition needs some explanation.

The bicuspids are the first to serve in the trituration of food. They have but one conical fang, whereas the molars have three above and two below. The solidity of these teeth is not very great, not to be compared with the molars.

Teeth have a movement in their sockets, and it is the more marked when they are less firm, less strongly held.

Well, then! It is one of two things; either the bicuspids of the subject have a hard enamel, or the enamel is slightly ossified, friable. If it is hard, they would wear away by rubbing, and a little flat facet will be found at the place of their contact. If it is friable, on the contrary, it would be ground in a measure from the movement in mastication and the point of contact will present a surface of a dull white color at first, if early examined, like the point of a transparent marble that has been forcibly struck. The enamel is here crushed, it has lost its molecular arrangement, its disintegration intensifies the energy of the acids which act upon it. Now alimentary detritus begins its work with the aid of the buccal mucus, which is in much greater quantity when the approximal surfaces of these teeth are flat in their whole extent, large and separated only above near the gum.

VI.—The first molars are oftener destroyed than the others, the wisdom tooth excepted.

The first molars are formed about the third year, and are erupted about the sixth. At the epoch of their formation the child is more lymphatic than in after years. The teeth too are not so well ossified, they are softer than those which form subsequently. Lastly, their grinding surface presents a vast number of furrows and eminences and the numerous funnels formed by these multiplied folds retain the alimentary detritus. They absorb readily and by such a number of points that it is not uncommon to see the grinder's surface blackened, and almost entire surface at once a prey to chemical destruction.

VII.—The molars (especially the superior) until now sound, often decompose by their approximal surface in mature age.

Projections of the gum, festoons occupy these interdentiary spaces. These festoons disappear at the time when the gums begin to recede and lay bare the teeth; their place is then occupied by aliments which remain there, ferment and decompose the approximal surfaces. These teeth are flat, very large, deprived of enamel above, near the neck where the ivory of the fangs is already exposed. Destruction may make great inroads before it is suspected and the individual complain of acute pain when the eye and the probe cannot yet fix the cause.

VIII.—The inferior molars are sometimes destroyed in old age by the external surface and near the neck.

This decomposition occurring in old persons is a singular form which has for a long time occupied our attention because it escaped a satisfactory explanation. This decomposition of the external surface near the neck is frequently met with on the inferior molars. They are excavated in such a manner that the crown is often nearly detached from the fangs, and the havoc seems to be arrested at the line where the enamel terminates whilst it keeps advancing in depth. The tooth appears as if sawn by a very thick saw.

What was the cause of this phenomenon? Why did chemical agents act here in so exceptional a manner? What were they? Questions often revolved in our mind and never solved. Finally we were put on the right path, and under these circumstances:

An old man whom we had known for many years, who had had excellent teeth all his life, a few years since lost an inferior molar every six months from the effect of this strange decomposition.

On questioning him about his health, on the medicaments he had employed which might occasion it, we learned that he was subject to a catarrhal affection of the throat of very frequent occurrence, that he made an almost habitual use of sweetened drinks. The sugar was a sufficient cause up to a certain point, but why did it destroy in preference the lower molars on the external surface near the neck?

We discovered after much questioning, gropingly directed and by one of those accidents of conversation which sometimes casts a ray of light, that he ordinarily kept a bit of lichen or other pastes in his mouth in order to diminish the cough he is afflicted with.

There was now no longer any doubt; he allowed these sugared and mucilaginous pastes to remain and melt slowly between the cheek and the neck of the inferior molars; their action was expended there and these teeth covered by solid enamel which had persisted until then, endowed with a resisting, well ossified tissue, only allowed themselves to be destroyed below the enamel, about the neck where the ivory was bare as is the case when the gums have receded.

Enlightened by this observation, we had the greatest desire, as one might readily imagine, to confirm it by other instances whenever an opportunity might offer. We now feel sure that the explanation of the fact being due to the presence of sugar, in all the so-called pectoral pastes, is perfectly correct. Our brethren who have frequent occasion to observe this can verify its truth.

IX.—The superior wisdom teeth are often attacked by their external surface, the inferior often by their grinding surface.

The superior wisdom teeth are often erupted with an inclination outwards toward the cheek. In this position their external surface often becomes nearly superior. The cheek rubs against the external border, causing aliments to accumulate upon this surface above the border, and it thus resembles, on account of its obliquity, the bottom ofany vessel. You can predict the consequences of such a disposition.

As to the inferior molars, they remain a long time beneath the gum, but only partially covered. Substances pass under this sort of cap, and when the grinding surface is fairly unmasked, it appears brown, infiltrated, decomposed; sometimes the tooth is nearly destroyed. It is one of the grossest errors to suppose that it was attacked before eruption, that is to say, before it had pierced the gum at any point whatsoever. Such an occurrence never takes place, for the simple reason that it is impossible.

X.—The inferior wisdom teeth decompose oftener than the superior.

The difficulty which these teeth experience in eruption, the length of time occupied in accomplishing it, the almost insurmountable obstacle which the ramus of the inferior maxilla opposes to them, retains them in a large number of cases, several months, several years in the worst physical conditions. During this period folds of inflamed gum surround them and form a kind of pit above their grinding surface which alone is visible; alimentary detritus can lodge there without the point of the tongue being able to dislodge it.

The superior wisdom tooth, on the contrary, advances more rapidly, and does not encounter any of these obstacles, any of these delays, and is less exposed to destruction than the inferior.

The applications of the theory which we have exposed can be multiplied; but the true cause of the decomposition of the teeth once known, it is easy for every intelligent man to explain the thousand and one modes in which this cause acts. Based upon it, every fact explains itself clearly and naturally. We therefore stop here, feeling assured that we have done enough by indicating the path.

### CONCLUSION.

The destruction of the teeth being due to chemical agents, as we have already shown, there are many ways of opposing these agents. 1. By preventing their formation. 2. By the administration of substances which neutralize them. 3. By placing the parts most liable to decompose in a more favorable condition.

The formation of the most active acids is prevented by not allowing the alimentary detritus to sojourn and ferment, by discontinuing the habitual use of aliments, drinks, or particular condiments which give origin to them.

Many substances retard, arrest putrid fermentation: others, alkaline, neutralize the action of the acids. Such may enter into the preparation of daily use, selecting them with care according to the subject and his particular condition. It is sometimes useful to employ them as a gargle, as a topical application, &c.

Various operations are also of great service, some are indispensable, before the employment of any other means. They should always be performed by a skillful hand, for any portion of the tooth once taken away is never reproduced, and time cannot restore the blunders committed by an unskillful practitioner.

But we propose as the subject of a second memoir, the examination and criticism of all the proper means for the preservation of the teeth and arresting their destruction. The absolute theory we have put forth as an exact expression of the truth, throws, you perceive, on all practical questions a new light, and ought to modify the means employed, the operations practiced up to the present time. There are some of them so unreasonable, so wanting in judgment, that we shall abandon them. We will submit something new to the intelligence of our worthy colleagues. We do not wish to be understood, however, to say that all which skillful practitioners have done and still do, is condemnable and injurious; this is far from our thoughts. Still, we avow that it must be very difficult for those who live in complete ignorance of the cause which destroys the teeth, to do just what is required, only what is required, and all as it should be done.

This important question with the development it admits of. But it was indispensable in the first place, and above all, to establish this truth, by which we will terminate, a truth, the results of which are so vast. The *destruction of the teeth is owing to a* CHEMICAL cause, *and not due to a* PATHOLOGICAL *cause, to a disease termed* CARIES.

*Science and art properly directed, offer numerous and efficacious resources to prevent, combat and arrest this destruction in its march.*

This last proposition will be, we repeat, the subject of a second memoir.—*N. Y. Dent. Rec.*

